# MiR-143-5p serves as a diagnostic biomarker in patients with sepsis and regulates sepsis-induced inflammation and cardiac dysfunction

**DOI:** 10.1186/s41065-025-00623-0

**Published:** 2025-12-10

**Authors:** Yaqi Wu, Le Gu, Xu Huang

**Affiliations:** 1 Department of Infection Management, Xuzhou Central Hospital, Southeast University, Xuzhou, 221000 China; 2 Department of Intensive Care Unit, Xuzhou Central Hospital, Southeast University, Xuzhou, 221000 China; 3 Department of Emergency, Xuzhou Central Hospital, Southeast University, No.199, Jiefang South Road, Xuzhou, 221000 China

**Keywords:** MiR-143-5p, Sepsis, Cardiac dysfunction, Inflammation

## Abstract

Background & Objective. MicroRNAs (miRNAs) offer advantages in stability and therapeutic specificity. This study investigated the diagnostic and therapeutic potential of miR-143-5p in sepsis (SP) and SP-associated cardiac dysfunction (CD). ‌Methods. Quantitative Real-Time polymerase chain reaction (qRT-PCR) quantified serum and cellular miR-143-5p levels. Receiver operator characteristic (ROC) curve evaluated miR-143-5p’s diagnostic efficacy. Pearson correlation analysis assessed the association between miR-143-5p and SP. Univariate logistic regression identified CD risk factors, with multivariate logistic analysis including significant variables from univariate analysis. Lipopolysaccharide (LPS)-induced macrophage (THP-1) and cardiomyocyte (AC16) models elucidated miR-143-5p mechanisms in SP, with bioinformatics predicting the potential pathways. ‌Results. MiR-143-5p downregulation demonstrated diagnostic value for SP (AUC: 0.897) and SP-CD (AUC: 0.812). MiR-143-5p expression correlated (*P* < 0.0001) with white blood cell count (WBC, *r* = -0.680), C-reactive protein (CRP, *r* = -0.563), procalcitonin (PCT, *r* = − 0.693), left ventricular ejection fraction (LVEF, *r* = 0.640), cardiac troponin I (cTnI, *r* = -0.599), Acute Physiology And Chronic Health Evaluation II (APACHE II, *r* = -0.695), and Sequential Organ Failure Assessment (SOFA, *P* < 0.05) scores. MiR-143-5p served as a risk factor for CD in SP (OR: 0.100). MiR-143-5p overexpression reduced M1 polarization and pro-inflammatory cytokines in LPS-treated THP-1. In AC16 cardiomyocytes, it enhanced viability, suppressed apoptosis, and attenuated inflammation. Bioinformatics analysis indicated miR-143-5p was involved in p53 and MAPK signal pathways regulation. ‌Conclusions. MiR-143-5p downregulation showed diagnostic potential for SP and SP with CD, correlating with disease severity and CD risk. Mechanistically, miR-143-5p overexpression mitigated macrophage and cardiomyocyte injury.

**Supplementary Information:**

The online version contains supplementary material available at 10.1186/s41065-025-00623-0.

## Introduction

 Sepsis (SP), a life-threatening organ dysfunction caused by a dysregulated host response to infection, remains a global health burden with an estimated 48.9 million cases and 11 million deaths annually [1]. Despite advances in critical care, mortality rates exceed 20% in severe cases, compounded by long-term complications and socioeconomic costs [2].

A critical yet underappreciated complication is SP-associated cardiac dysfunction (CD), occurring in 40–60% of septic patients and correlating with mortality rates exceeding 70% [3]. CD induced by SP manifests as reduced ejection fraction, arrhythmias, or hemodynamic instability in 40% of patients [4]. The main pathomechanism of SP-induced CD presents as systemic inflammation-triggered cardiomyocyte injury, mitochondrial dysfunction, calcium dysregulation, and microcirculatory disturbances [5].

Current clinical management of SP prioritizes early antimicrobial therapy, hemodynamic stabilization via fluid resuscitation and vasopressors, and organ support [6]. However, these approaches fail to directly address the underlying molecular mechanisms driving myocardial injury, leaving many patients with persistent cardiac impairment even after SP resolution. The above therapeutic gap underscores the urgent need to identify novel molecular regulators of CD in SP.

MicroRNAs (miRNAs), small non-coding RNAs that post-transcriptionally regulate gene expression, have emerged as pivotal modulators of pathological processes due to their ability to fine-tune multiple signaling pathways simultaneously. Their high stability in bodily fluids, tissue-specific expression patterns, and dynamic responsiveness to cellular stress make them ideal biomarkers and therapeutic targets [7].

In SP, a hyperinflammatory state, miR-155 promotes pro-inflammatory cytokine release while miR-21 suppresses immune cell apoptosis [8, 9]. Notably, miRNA dysregulation is also involved in SP-associated CD. For instance, miR-208a-5p exacerbates cardiomyocyte injury by targeting XIAP, whereas miR-146a attenuates inflammation via Toll-like receptor pathways [10, 11]. Circulating miR-501-5p also shows potential value for early CD diagnosis in SP [12]. These studies indicate dysregulated miRNAs contribute to SP pathogenesis and cardiac injury, representing potential diagnostic biomarkers and therapeutic targets for improving sepsis clinical treatment and outcomes.

Emerging evidence highlights that miR-143-5p modulates macrophage polarization by suppressing pro-inflammatory cytokines and promoting M2 phenotype transition in abdominal aortic aneurysm, suggesting its regulatory role in SP-associated hyperinflammation [13]. MiR-143-5p upregulation has also been shown to attenuate SP-induced angiosteosis linking to inflammation and immunity [14]. In addition, dysregulated miR-143-5p expression is found in SP and SP-associated CD according to the online network database in a previous study [15]. MiR-143-5p was also involved in atherosclerosis, the foundation of acute myocardial infarction and heart failure, underscoring its connection to cardiac function [16].

Based on the above evidence, it could be hypothesized that miR-143-5p might be involved in SP and SP-associated CD progression through regulating macrophage polarization and myocardial cell function, which remains to be confirmed through clinical or in vitro/vivo experiments. Thus, the present study was conducted to investigate the clinical significance of miR-143-5p in SP and SP-induced CD, in addition to its potential mechanisms of action in SP progression. The findings may offer novel insights into the clinical management of SP.

## Subjects and methods

### Subjects

#### Selection of clinical subjects

This study enrolled 189 sepsis (SP) patients who were diagnosed according to the criteria outlined in the Third International Consensus Definitions for SP and Septic Shock published in 2016 [17], and hospitalized in the Xuzhou Central Hospital, Southeast University as the research cohort. SP patients were stratified into two groups: normal cardiac function group (Non-CD, *n* = 94) and cardiac dysfunction group (CD group, *n* = 95) according to the results of the electrocardiograph and ultrasonic cardiogram. A control group comprising 77 healthy individuals undergoing routine physical examinations at the same hospital during the study period was established. Sample size calculation was performed using G*Power 3.1, with an effect size of 0.5, α error probability of 0.05, and power of 0.80, indicating a requirement of 64 samples per group. To account for potential sample loss and other factors that might compromise data validity, we enrolled 77 healthy volunteers and 189 sepsis subjects, which yielded an achieved power of 0.96 based on post-hoc analysis. Additionally, the sample sizes in the subgroups (CD and non-CD groups) achieved a power of 0.93. All subjects or their families had signed the informed consent, and this study was approved by the Ethics Committee of the Xuzhou Central Hospital, Southeast University.

The inclusion criteria of the SP subjects: (1) The age of subjects was more than 18 years old and less than 80 years old; (2) The relevant clinical data of the subjects were complete.

The exclusion criteria of the SP subjects: (1) Immunodeficiency disorders‌; (2) Severe hepatic or renal insufficiency; (3) History of malignant tumors, (4) Mental disease, (5) Accepted treatment with corticosteroids and immunosuppressive drugs, 5) ‌During pregnancy and lactation.

### Cells

Human leukemic monocytic cell line THP-1 (ATCC, USA).

Human myocardial cell line AC16 (ATCC, USA).

### Methods

#### Clinical data collection and blood sample processing

The clinical information, including age, gender, and body mass index (BMI), of all subjects was collected within 24 h of hospitalization. The fasting blood samples of all subjects were collected on the morning of the next day after the hospitalization and used for the analysis of the biochemical indexes such as white blood cell count (WBC), C-reactive protein (CRP), procalcitonin (PCT), cardiac troponin I (cTnI) levels, etc., which were analyzed by the fully automatic biochemical analyzer. The SP scoring system, including severity-Acute Physiology and chronic health evaluation II (APACHE II) and organ dysfunction-Sequential Organ Failure Assessment (SOFA) scores, was also evaluated by the criteria of the scoring system [18, 19]. Serum was collected from the fasting blood samples of all subjects after centrifugation at 3700 rpm for 10 min at 4 ℃ and then stored at −80 ℃ for further analysis.

#### Cell culture and cell model conduction

THP-1 cells were used to mimic the early inflammatory events triggered by bacterial infection in SP. THP-1 monocytes were maintained in RPMI-1640 medium (Life Technologies, USA) supplemented with 10% fetal bovine serum (FBS, Gibco, USA) and 1% penicillin/streptomycin (P/S, Gibco, USA) at 37 °C under 5% CO₂. AC16 cardiomyocytes were cultured in a DMEM medium (Thermo Fisher Scientific, USA) containing 10% FBS (Gibco, USA) and 1% P/S (Gibco, USA) under the same culture conditions as THP-1. For LPS stimulation, cells were seeded in 6-well plates (1 × 10^5^ cells/well). When the cell confluence reached 70%, cells were treated with LPS (20 ng/mL for THP-1 and 1 µg/mL for AC16, Sigma-Aldrich, USA) for 24 h [20, 21]. Post-treatment, the supernatant of the cells was collected. All experiments included at least three technical replicates.

#### Cell transfection

The overexpression of miR-143-5p in THP-1 and AC16 was conducted by the miR-143-5p mimic (Ribobio, China). After the LPS induction, the miR-143-5p mimic (50 nM) and its negative control were transfected into the THP-1 and AC16 cells (2 × 10^5^ cells/well) with the help of Lipofectamine 2000 (Invitrogen, USA) to model a therapeutic intervention after the onset of inflammatory response [22, 23]. Further incubated for 48 h, the cells were collected for the downstream analysis.

#### qRT-PCR

Total RNA of the serum and cell samples was extracted with the help of the Trizol reagent (Sigma-Aldrich, USA), and then the RNA quality was verified via NanoDrop (A260/A280 > 1.8). The extracted RNA was transformed into cDNA by using the PrimeScript RT Kit (TaKaRa, Japan). Quantitative real-time PCR was conducted using SYBR Green Master Mix (Applied Biosystems, USA) on an ABI PRISM 7300 system (Thermo Fisher Scientific, USA) with the following thermal profile: 95 °C for 10 min, 40 cycles of 95 °C for 15 s and 60 °C for 1 min. The expression levels of miR-143-5p, M1 polarization markers (inducible nitric oxide synthase: iNOS and cluster of differentiation 86: CD86), and M2 polarization markers (Arginase-1: Arg-1 and cluster of differentiation 206: CD206) were calculated by the 2^‑△△Ct^ method with cel-miR-39 and GAPDH serving as the internal control. The primer sequences were listed in Table 1.

**Table 1:** The primer sequences used in the study

Primer	Forward	Reverse
miR-143-5p	5’-GGTGCAGTGCTGCATCT-3’	5’-CTCAACTGGTGTCGTGGA-3’
iNOS	5’-GCAGGACTCACAGCCTTTGG-3’	5’-GGCTGGATGTCGGACTTTGT-3’
CD86	5’-GTTTCATTCCCTGATGTTACGAG-3’	5’-GAGAAAGGTGAAGATAAAAGCCG-3’
Arg-1	5’-GGAAGTGAACCCATCCCT-3’	5’-GATTACCCTCCCGAGCA-3’
CD206	5’-GATGATACCTGCGACAGTAAACG-3’	5’-GCTTGCAGTATGTCTCCGCTT-3’
cel-miR-39	5’-UCACCGGGUGUAAAUCAGCUUG-3’	5’-TCACCGGGTGTAAATCAGCTTG-3’
GAPDH	5’-GGGGCTCTCCAGAACATC-3’	5’-TGACACGTTGGCAGTGG-3’

*iNOS* inducible nitric oxide synthase, *CD86* cluster of differentiation 86, *Arg-1* Arginase-1, *CD206* cluster of differentiation 206

#### CCK-8 (Cell counting kit-8) and flow cytometry (FCM)

The cells were seeded into 96-well plates at a density of 2 × 10^3^ cells per well after treatment with experimental conditions and cultured for 24–72 h. Then 10 µL of CCK-8 reagent (Dojindo, Japan) was added to each well, followed by incubation for 2 h at 37 °C. The optical density (OD) at 450 nm was measured using a microplate reader (BioTek Synergy H1, USA).

The AC16 cells were cultured in 6-well plates (2 × 10^5^ cells/well) and subjected to experimental treatments. Post-intervention, cells were washed twice with ice-cold PBS and centrifuged (1000 rpm, 5 min). The cell pellet was gently resuspended in 200 µL binding buffer. Subsequently, 5 µL Annexin V-FITC (Sigma-Aldrich, USA) was added and incubated for 15 min at 4 °C in the dark, followed by the addition of 10 µL propidium iodide (PI, Sigma-Aldrich, USA) and incubation for 5 min. Cellular apoptosis was analyzed immediately using a flow cytometer (BD FACSCanto II).

#### Enzyme-linked immunosorbent assay (ELISA)

The level of inflammatory cytokines (tumor necrosis factor-α: TNF-α and interleukin-6: IL-6) was assessed and quantified using human ELISA kits (EZHTNFA-150 K/EZIL6, Sigma-Aldrich, USA). After different experiment treatments, the supernatant of the cells (2 × 10^5^ cells/well, 6-well plate) was collected and added to the 96-well plate. Detection antibodies, streptavidin-HRP, and TMB substrate were sequentially added following the manufacturer’s instructions. The absorbance (450 nm) was measured within 15 min, and concentrations were calculated.

#### Bioinformatic analysis

The downstream targets of miR-143-5p were predicted using online databases, including miRDB (https://mirdb.org/) and Target Scan Human (https://www.targetscan.org/vert_72/). Overlapping genes among these predictions were analyzed and visualized using a Venn diagram. Potential signaling pathways that these target genes might be involved in were further predicted through the DAVID Functional Annotation Tools (https://davidbioinformatics.nih.gov/).

### Statistical analysis

The data processing was performed by GraphPad 9.0 and SPSS. All data were presented by the mean value ± SD. The P-P plot is used to evaluate the normal distribution. The difference between the two groups was analyzed by the Student’s t-test, and one-way ANOVA with post hoc Tukey’s test was used for the multiple groups comparison. Diagnostic potential of miR-143-5p on SP and SP-induced CD was evaluated through the receiver operator characteristic (ROC) curve. The association between miR-143-5p and SP severity was analyzed by the Pearson correlation analysis, and the risk factors for SP-associated CD among SP subjects were assessed by the univariate Logistic regression, with variables showing a p-value < 0.05 further included in the multivariable Logistic regression model to identify independent risk factors. The significance was represented as *P* < 0.05.

## Results

### Clinical data comparison

The clinical characteristics of the study cohorts were summarized in Table 2. No significant intergroup differences were observed in baseline demographics, including age, gender distribution, BMI, or infection sites (*P* > 0.05)‌. Compared to healthy controls, SP subjects (both with and without CD) demonstrated significantly decreased PLT (*P* < 0.001), elevated inflammatory markers (WBC: *P* < 0.001; CRP: *P* < 0.001; PCT: *P* < 0.001), increased Lac (*P* < 0.001), impaired liver and kidney functions evidenced by the ascending Scr (*P* < 0.001), TB (*P* < 0.001), ALT (*P* < 0.001), and AST (*P* < 0.001) levels, while LVEF showed no significant difference between non-CD subjects and healthy controls (*P* = 0.078)‌. Notably, CD subjects exhibited significantly elevated WBC (*P* = 0.002), CRP (*P* = 0.041), PCT (*P* = 0.005), cTnI (*P* < 0.001), and APACHE II scores (*P* < 0.001), along with reduced PLT (*P* = 0.002) and LVEF (*P* < 0.001) compared to non-CD counterparts‌. Furthermore, CD subjects showed greater disease severity as evidenced by higher SOFA scores (≥ 2 in 55.79% vs. 37.23%, *P* = 0.011)‌.

**Table 2:** Clinical data of the subjects

Indicators			HC group (*n* = 77)	Non-CD group (*n* = 94)	CD group (*n* = 95)	*P*_1_ value	*P*_2_ value	*P*_3_ value
Age (year)			54.78 ± 7.38	56.69 ± 9.14	55.64 ± 6.96	0.259	0.757	0.633
Gender		Male	51 (66.23%)	59 (62.77%)	62 (65.26%)	0.638	0.894	0.721
		Female	26 (33.77%)	35 (37.23%)	33 (34.74%)			
BMI (kg/m^2^)			22.26 ± 1.43	22.20 ± 0.99	22.61 ± 1.69	0.963	0.232	0.114
Infection site	Enterocoelia		-	34	29	-	-	0.528
	Bloodstream			17	25			
	Respiratory system			19	16			
	Skin and soft tissue			11	15			
	Other			13	10			
WBC (×10^9^/L)			7.15 ± 0.78	15.04 ± 2.55	16.28 ± 3.19	< 0.001	< 0.001	0.002
PLT (×10^9^/L)			172.81 ± 25.77	129.74 ± 25.04	116.85 ± 27.72	< 0.001	< 0.001	0.002
CRP (mg/L)			2.71 ± 0.52	90.93 ± 9.76	95.43 ± 18.91	< 0.001	< 0.001	0.041
PCT (ng/mL)			0.02 ± 0.01	8.23 ± 2.81	9.36 ± 2.92	< 0.001	< 0.001	0.005
Lac (mmol/L)			1.11 ± 0.23	2.30 ± 0.42	2.43 ± 0.45	< 0.001	< 0.001	0.051
Scr (mg/dL)			0.82 ± 0.15	1.40 ± 0.45	1.51 ± 0.55	< 0.001	< 0.001	0.176
TB (µmol/L)			9.11 ± 1.69	13.64 ± 3.29	14.00 ± 3.90	< 0.001	< 0.001	0.717
ALT (U/L)			29.12 ± 4.93	49.43 ± 7.07	51.33 ± 9.19	< 0.001	< 0.001	0.182
AST (U/L)			30.47 ± 4.77	62.40 ± 9.78	65.63 ± 12.62	< 0.001	< 0.001	0.065
LVEF (%)			55.09 ± 5.30	52.55 ± 7.12	48.03 ± 9.40	0.078	< 0.001	< 0.001
cTnI (µg/L)			-	0.93 ± 0.14	1.07 ± 0.27	-	-	< 0.001
APACH II score			-	15.99 ± 2.48	17.33 ± 2.90	-	-	< 0.001
SOFA score		< 2	-	59 (62.77%)	42 (44.21%)	-	-	0.011
		≥ 2	-	35 (37.23%)	53 (55.79%)			

Continuous variables were presented as mean ± SD

P_1_: HC vs. Non-CD, P_2_: HC vs. CD, P_3_: Non-CD vs. CD

*HC* Healthy Control, *Non-CD* Sepsis without Cardiac Dysfunction, *CD* Cardiac Dysfunction, *BMI* Body Mass Index, *WBC* White Blood Cell Count, *PLT* Platelet count, *CRP* C-Reactive Protein, *PCT* Procalcitonin, *Lac* Lactic acid, *Scr* Serum creatinine, *TB* Total bilirubin, *ALT* Alanine transaminase, *AST* Aspartate aminotransferase, *LVEF* ‌Left Ventricular Ejection Fraction, *cTnI* Cardiac Troponin I, *APACH* Acute Physiology and Chronic Health Evaluation, *SOFA* Sequential Organ Failure Assessment.

### The miR-143-5p expression in SP and the ROC curve

**Fig. 1:**
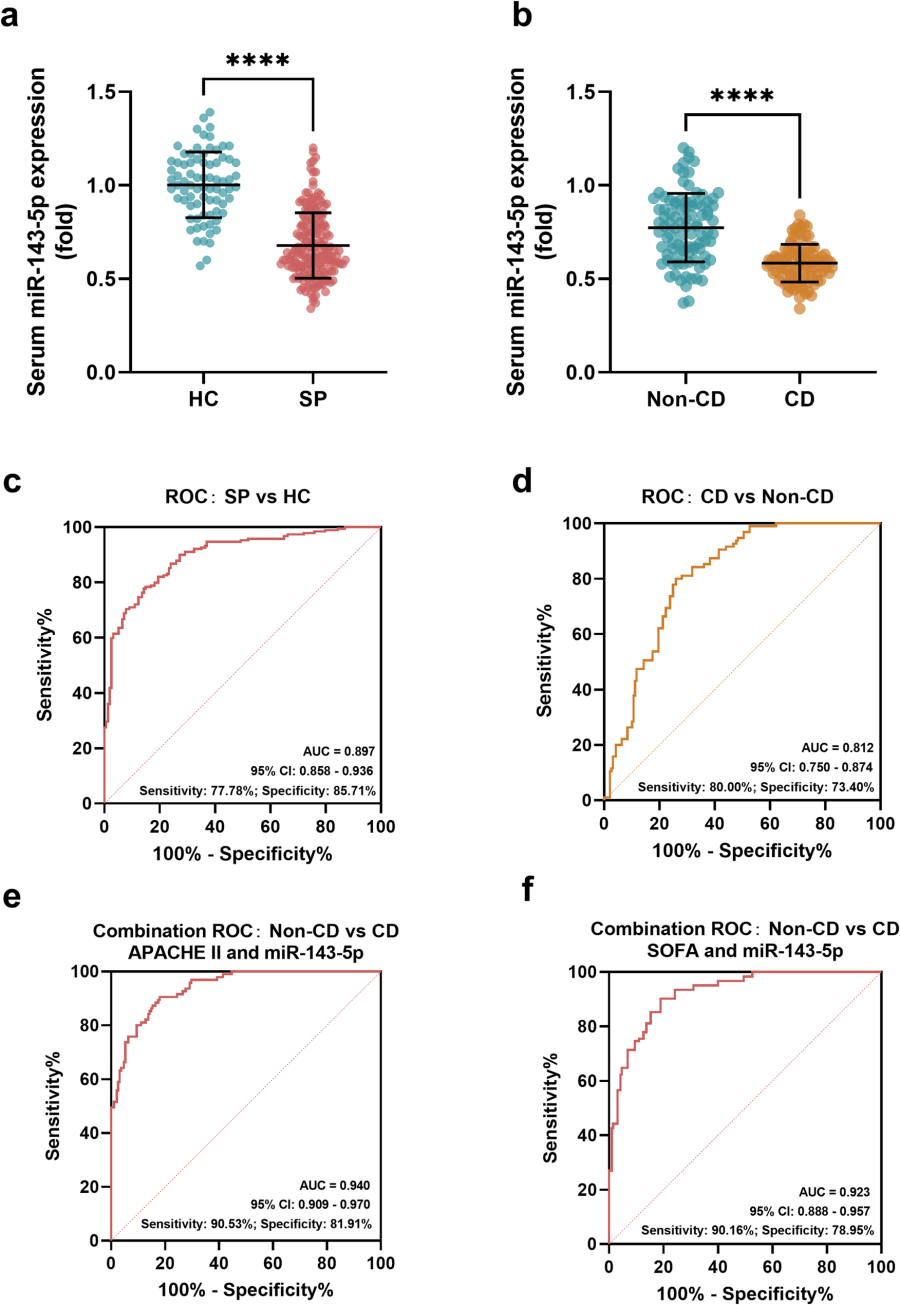
The serum miR-143-5p expression and ROC curve. **a**-**b** The miR-143-5p expression was (**a**) downregulated in SP compared with healthy individuals and (**b**) lower in SP-associated CD compared with SP without CD. **c** Downregulated miR-143-5p difference SP from healthy with the AUC of 0.897. **d** miR-143-5p downregulation distinguished SP-induced CD from SP with an AUC of 0.812. **e**-**f** Combination ROC Analysis of miR-143-5p combined with (**e**) APACHE II and (**f**) SOFA scores for diagnosing CD in SP. ****P < 0.0001. Statistical tests: Student’s t-test and ROC analysis

The miR-143-5p expression of the subjects and its diagnostic value in SP were revealed in Fig. 1. The serum miR-143-5p expression of SP was downregulated in comparison to that of the healthy control group (*P* < 0.0001, Fig. 1a). The SP subjects could be further divided into a non-CD group and a CD group according to whether the SP subjects developed a dysfunction of the heart. The miR-143-5p level of the SP subjects with CD was lower than that of the SP subjects without the CD (*P* < 0.0001, Fig. 1b). The diagnostic value of miR-143-5p was illustrated, and the miR-143-5p could differentiate SP subjects from healthy individuals with an area under the curve (AUC) of 0.897, sensitivity of 77.78%, and specificity of 85.71% (95% CI: 0.858–0.936, Fig. 1c) at the cut-off value of 0.805. Additionally, miR-143-5p could also distinguish the CD occurrence of SP subjects from those who didn’t develop the CD (cut-off: 0.655, AUC: 0.812, 95% CI: 0.750–0.874, sensitivity: 80.00%, specificity: 73.40%, Fig. 1d). Further combined ROC curve analyses revealed that the combination of miR-143-5p with APACHE II score (Fig. 1e) yielded an AUC of 0.940 (sensitivity: 90.53%, specificity: 81.91%), while the combination with SOFA score (Fig. 1f) achieved an AUC of 0.923 (sensitivity: 90.16%, specificity: 78.95%). These findings demonstrated that both combined panels exhibit superior diagnostic potential for CD among SP subjects compared to miR-143-5p alone.

### The association between miR-143-5p and SP severity

**Fig. 2:**
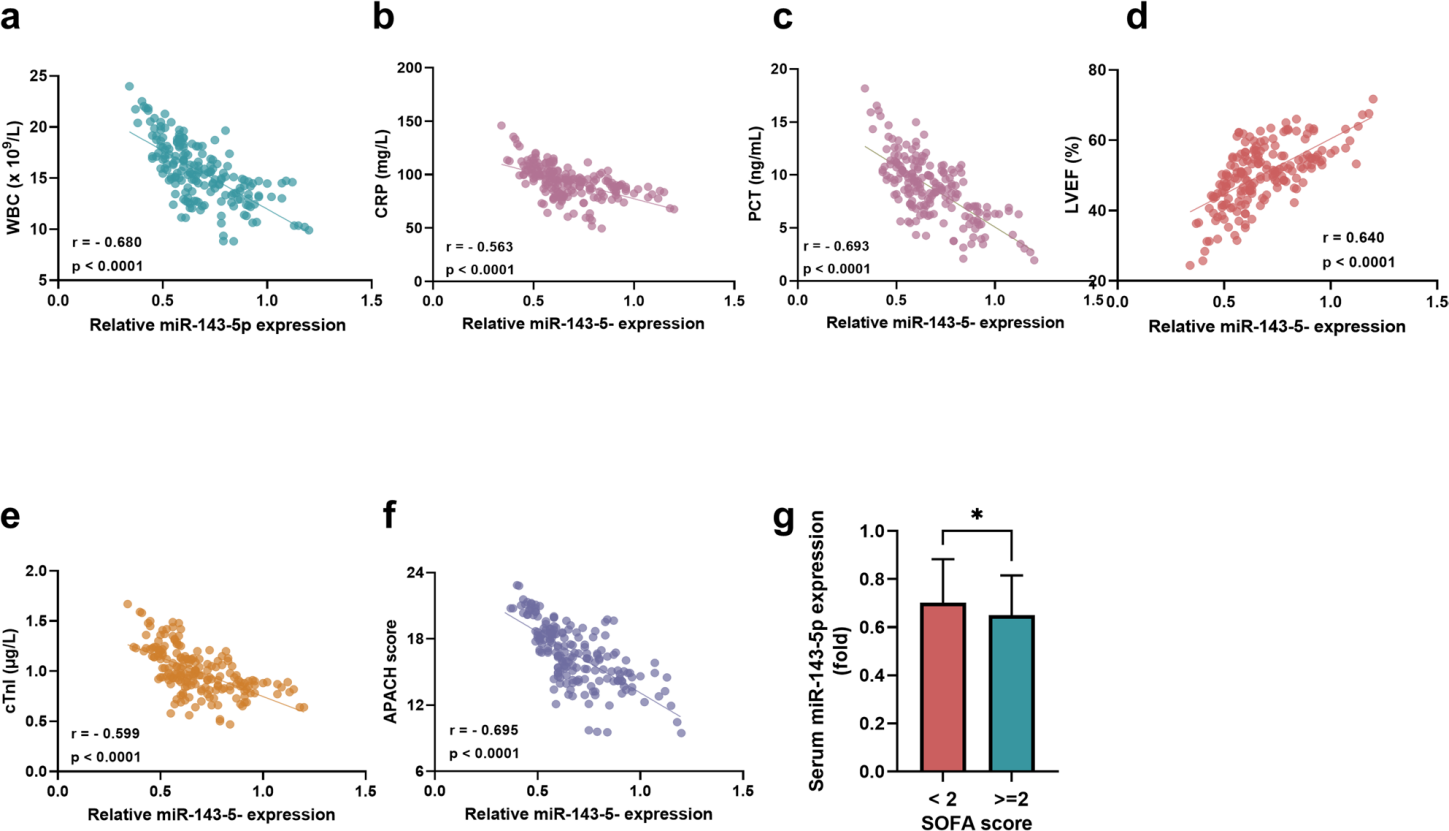
The miR-143-5p expression in SP was correlated with inflammation and cardiac function. **a-b** The miR-143-5p was negatively correlated with (**a**) WBC, (**b**) CRP, (**c**) PCT, (**e**) cTnI, (**f**) APACHE II score, and positively correlated with (**d**) LVEF. **g** Levels of miR-143-5p were significantly lower in SP subjects with SOFA scores ≥ 2 compared to those with scores < 2. *P < 0.05. Statistical tests: Pearson correlation analysis and Student’s t-test

A significant association between miR-143-5p expression and SP severity was demonstrated in Fig. 2. MiR-143-5p levels exhibited a strong inverse correlation with inflammatory markers in SP subjects, including WBC (*r* = −0.680, *P* < 0.0001, Fig. 2a), CRP (*r* = −0.563, *P* < 0.0001, Fig. 2b), and PCT (*r* = −0.693, *P* < 0.0001, Fig. 2c). Cardiac function parameters also showed a close correlation with miR-143-5p expression, with positive correlations observed for LVEF (*r* = 0.640, *P* < 0.0001, Fig. 2d) and negative correlations for cTnI (*r* = −0.599, *P* < 0.0001, Fig. 2e), indicating that miR-143-5p was closely associated with the ability of the left ventricle contracting and myocardial injury, and may play a role in SP-induced cardiac impairment. Furthermore, miR-143-5p expression negatively correlated with disease severity scores, including the APACHE II score (*r* = −0.695, *P* < 0.0001, Fig. 2f) and SOFA score stratification (lower expression in SP subjects with SOFA ≥ 2 vs. < 2, *P* < 0.05, Fig. 2g), both of which were validated scoring system for assessing SP severity and organ dysfunction degree, with higher scores indicating more severe illness and worse prognosis.

### Risk factors of SP-induced CD

The risk factors for CD occurrence in SP were firstly evaluated through univariate analysis (Table 3), and then the significant indicators were further validated by the multivariable Logistic regression analysis (Table 4). MiR-143-5p upregulation was identified as the risk factor for CD of SP subjects with the OR value of 0.100 (95% CI: 0.033–0.304, *P* < 0.001). In addition, the level of LVEF (OR: 0.410, 95% CI: 0.170–0.989, *P* = 0.047), cTnI (OR: 2.598, 95% CI: 1.144–5.899, *P* = 0.023), APACHE (OR: 2.654, 95% CI: 1.032–6.827, *P* = 0.043), and SOFA score (OR: 7.459, 95% CI: 2.789–19.945, *P* < 0.001) were also confirmed to serve as the risk factors for the incidence of CD in SP.

**Table 3:** The logistic regression of the risk factors for SP-induced CD among SP

Variate	Univariate analysis					
	β	SE	OR	95% CI		*P* value
Age	0.276	0.293	1.318	0.742	2.339	0.346
BMI	0.453	0.294	1.573	0.884	2.802	0.124
Gender	0.108	0.303	1.115	0.615	2.019	0.721
**WBC**	0.784	0.298	2.190	1.220	3.930	**0.009**
**CRP**	0.749	0.296	2.115	1.184	3.780	**0.011**
**LEVF**	−0.843	0.298	0.430	0.240	0.772	**0.005**
**cTnI**	1.358	0.310	3.890	2.118	7.145	**< 0.001**
**APACHE II**	0.926	0.299	2.526	1.406	4.536	**0.002**
**SOFA**	0.755	0.297	2.127	1.189	3.807	**0.011**
**miR-143-5p**	−2.335	0.356	0.097	0.048	0.194	**< 0.001**

The OR values of the univariate Logistic regression analysis were presented as unadjusted

*SP* Sepsis, *CD* Cardiac Dysfunction, *SE* Standard error, *OR* Odds ratio, *BMI* Body Mass Index, *WBC* White Blood Cell Count, *CRP* C-Reactive Protein, *LVEF* ‌Left Ventricular Ejection Fraction, *cTnI* Cardiac Troponin I, *APACHE* Acute Physiology and Chronic Health Evaluation, *SOFA* Sequential Organ Failure Assessment

**Table 4:** The multivariate logistic regression of the risk factors for SP-induced CD among SP

Variate	Multivariate analysis					
	β	SE	OR	95% CI		*P* value
WBC	0.725	0.459	2.065	0.840	5.075	0.114
CRP	0.432	0.424	1.541	0.671	3.535	0.308
**LEVF**	−0.892	0.449	0.410	0.170	0.989	**0.047**
**cTnI**	0.955	0.419	2.598	1.144	5.899	**0.023**
**APACHE II**	0.976	0.482	2.654	1.032	6.827	**0.043**
**SOFA**	2.009	0.502	7.459	2.789	19.945	**< 0.001**
**miR-143-5p**	−2.307	0.569	0.100	0.033	0.304	**< 0.001**

The OR values of the multivariate analysis were presented as adjusted according to the univariate Logistic regression analysis

*SP* Sepsis, *CD* Cardiac Dysfunction, *SE* Standard error, *OR* Odds ratio, *WBC* White Blood Cell Count, *CRP* C-Reactive Protein, *LVEF* ‌Left Ventricular Ejection Fraction, *cTnI* Cardiac Troponin I, *APACHE II* Acute Physiology and Chronic Health Evaluation II, *SOFA* Sequential Organ Failure Assessment

### Regulatory effect of miR-143-5p in macrophage and myocardial cell injury, and the bioinformatic analysis

**Fig. 3:**
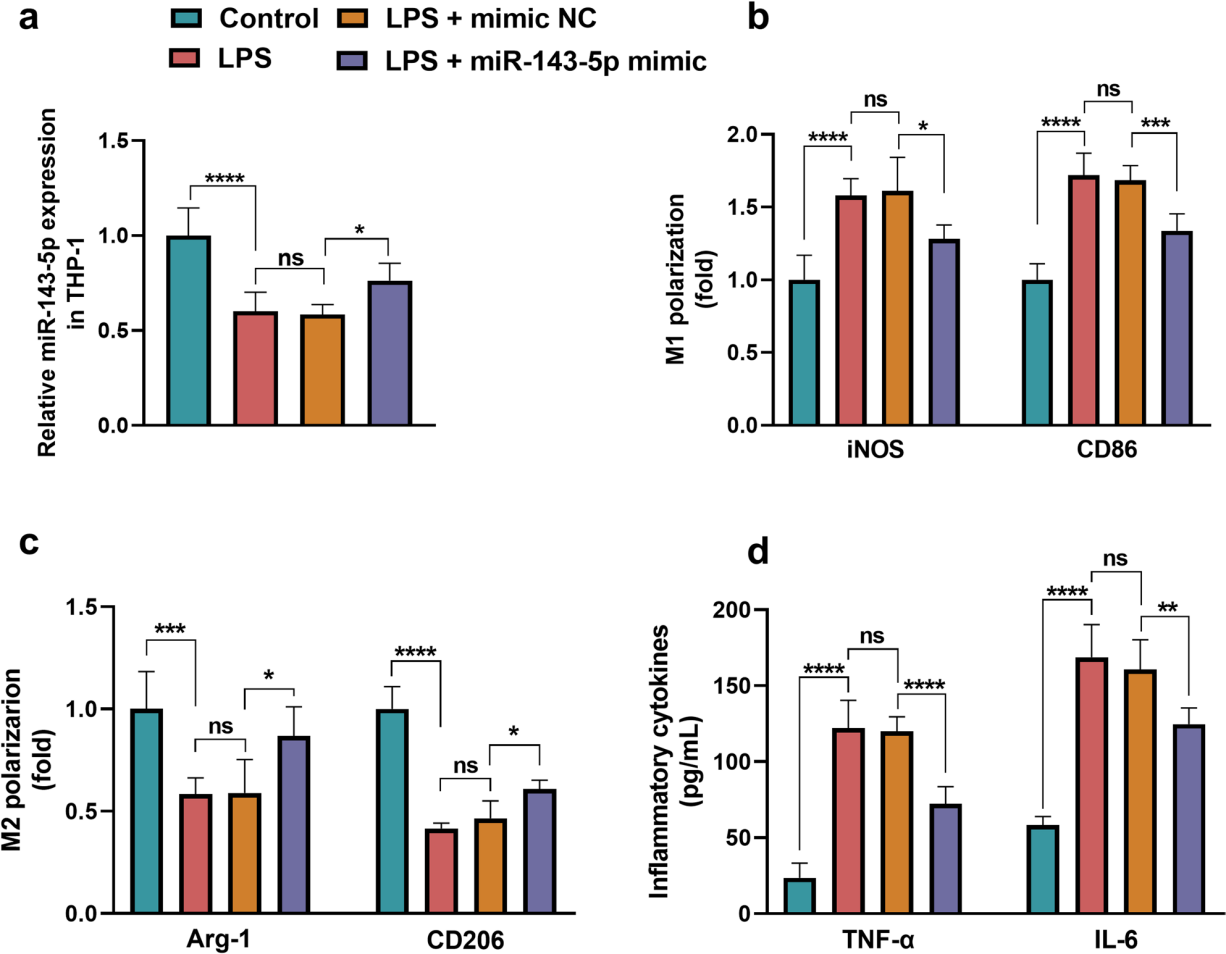
The effect of miR-143-5p on LPS-induced THP-1. **a** The miR-143-5p expression was downregulated in LPS-induced THP-1 and could be upregulated by miR-143-5p mimic transfection. **b**-**c** Overexpression of miR-143-5p suppressed the level of (**b**) M1 polarization markers (iNOS and CD86) and improved the (**c**) M2 polarization markers (Arg-1 and CD206) expression in LPS-induced THP-1. **d** MiR-143-5p upregulation inhibited the level of inflammatory cytokines, including TNF-α and IL-6, in THP-1 induced by LPS. ns: no significance, *P < 0.05, **P < 0.01, ***P < 0.001, ****P < 0.0001. Statistical tests: one-way ANOVA with post hoc Tukey’s test

After the induction of LPS in THP-1, the expression of miR-143-5p was decreased (*P* < 0.05) and could be upregulated by the transfection of miR-143-5p mimic (*P* < 0.0001, Fig. 3a). The level of M1 polarization markers, including iNOS (*P* < 0.0001) and CD86 (*P* < 0.0001), was observed to be increased (Fig. 3b) while the M2 polarization markers, including Arg-1 (*P* < 0.001) and CD206 (*P* < 0.0001), were decreased (Fig. 3c) in LPS-induced THP-1. The inflammatory cytokines, including TNF-α (*P* < 0.0001) and IL-6 (*P* < 0.0001), were also elevated in LPS-stimulated THP-1 (Fig. 3d), suggesting the successful induction of LPS-induced THP-1 polarization and inflammation. Overexpression of miR-143-5p exhibited contrasting effects on LPS-induced THP-1 cell polarization and inflammation, significantly suppressing the iNOS (*P* < 0.05) and CD86 (*P* < 0.001) expression, while facilitating the Arg-1 and CD206 expression (*P* < 0.05), also inhibiting the levels of TNF-α (*P* < 0.0001) and IL-6 (*P* < 0.01).

**Fig. 4:**
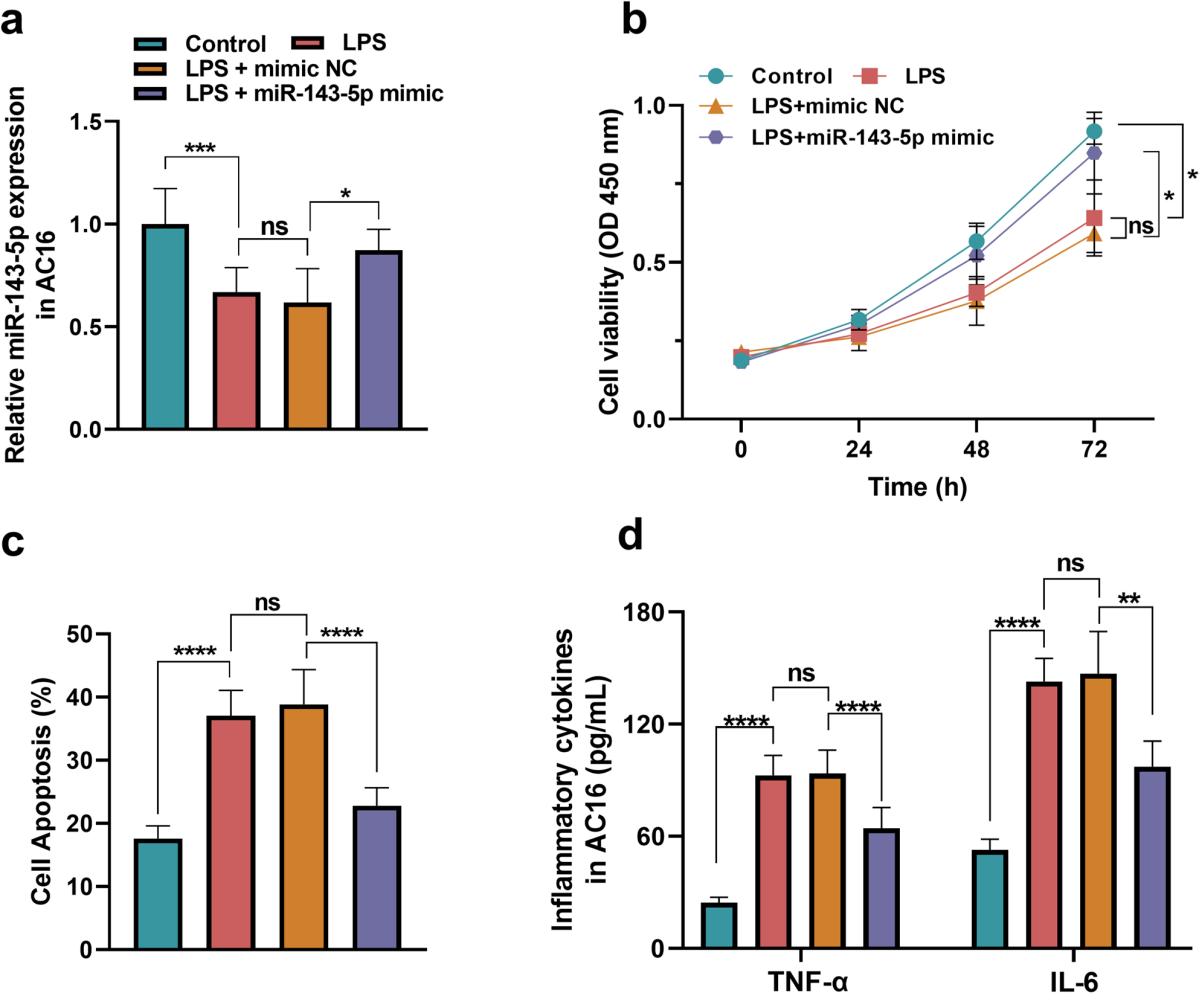
The effect of miR-143-5p on LPS-induced AC16 injury. **a** The miR-143-5p was downregulated in LPS-induced AC16. **b**-**d** Overexpressed miR-143-5p could promote (**b**) the cell viability, suppress (**c**) the apoptosis, and (**d**) the inflammatory cytokines (TNF-α, IL-6) of LPS-induced AC16. ns: no significance, *P < 0.05, **P < 0.01, ***P < 0.001, ****P < 0.0001. Statistical tests: one-way ANOVA with post hoc Tukey’s test

The miR-143-5p expression was downregulated (*P* < 0.001) in the LPS-induced AC16, and overexpression of miR-143-5p after LPS induction in AC16 cells significantly modulated miR-143-5p expression levels (*P* < 0.05, Fig. 4a). LPS stimulation could also suppress cell viability (*P* < 0.05, Fig. 4b), facilitate cell apoptosis (*P* < 0.0001, Fig. 4c), and promote inflammatory responses (TNF-α: *P* < 0.0001; IL-6: *P* < 0.0001, Fig. 4d) in AC16, demonstrating the impaired AC16 cell function. However, miR-143-5p upregulation showed a reverse effect on the cell viability, apoptosis, and inflammation in LPS-induced AC16. Specifically, overexpressed miR-143-5p level promoted the cell viability (*P* < 0.05) that had been suppressed by LPS and inhibited cell apoptosis (*P* < 0.0001). Additionally, the elevated miR-143-5p level could also reduce the level of TNF-α (*P* < 0.0001) and IL-6 (*P* < 0.01) in the AC16 induced by LPS.

**Fig. 5:**
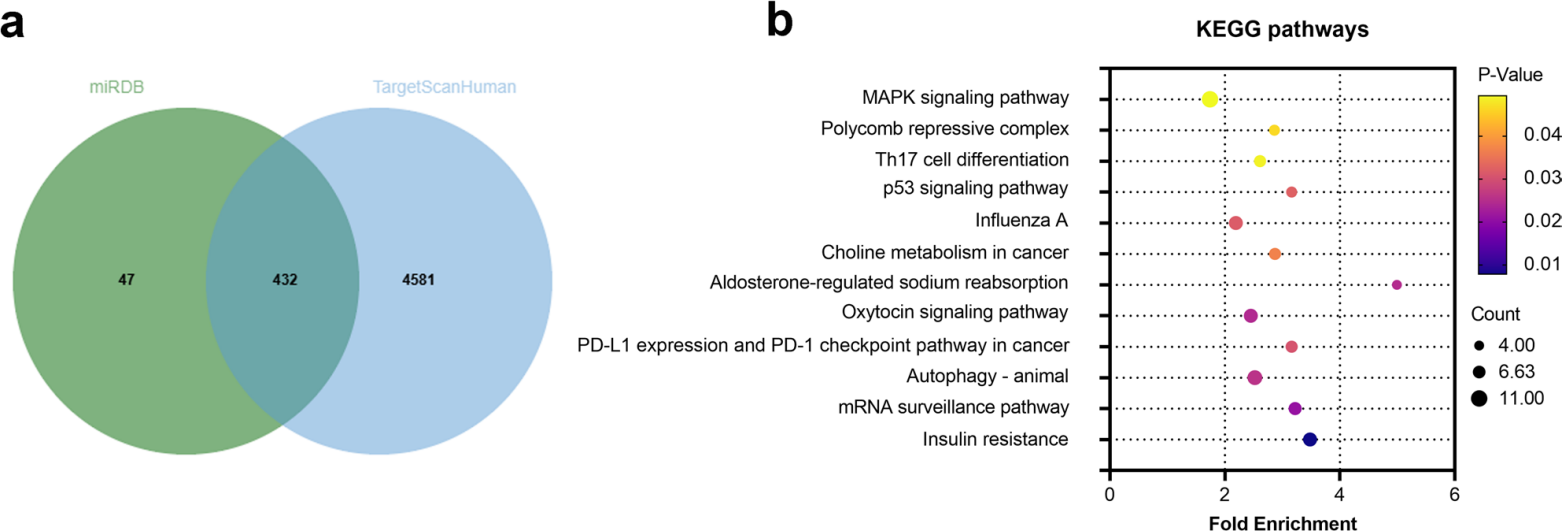
Bioinformatic analysis of miR-143-5p downstream targets and potential pathways. **a** Venn diagram of the overlapped genes of miR-143-5p predicted by the online databases miRDB and TargetScanHuman. **b** KEGG pathway analysis of the 432 overlapping genes

Further bioinformatics analysis indicated that there was a total of 432 overlapping target genes of miR-143-5p (Fig. 5a). Kyoto Encyclopedia of Genes and Genomes (KEGG) pathway analysis (Fig. 5b) revealed that these genes are involved in regulating the mRNA surveillance pathway, p53 signaling pathway, and MAPK signaling pathway. Given that the p53 pathway is a well-established regulator of cell apoptosis and the MAPK pathway is critical for inflammation modulation, these findings strongly suggest that miR-143-5p may exert its biological effects by orchestrating the regulation of both apoptotic and inflammatory processes through these signaling cascades. This regulatory mechanism potentially provided a molecular basis for the role of miR-143-5p in modulating THP-1 macrophage polarization, AC16 cell viability, and inflammatory responses.

## Discussion

MiRNAs have emerged as promising candidates for disease diagnosis and treatment due to their stability in biofluids, tissue-specific expression profiles, and capacity to regulate disease-associated pathways [24]. MiR-143-5p exemplified this multifunctionality, with reported roles in diabetic retinopathy (via angiogenesis modulation) [25], idiopathic pulmonary fibrosis (through TGF-β/Smad3 signaling) [26], and gastric cancer metastasis (by cytoskeletal remodeling) [27]. This study evaluated the clinical significance of miR-143-5p in SP and its possible role in regulating the injury of macrophages and cardiomyocytes.

Our clinical analysis delineated miR-143-5p as a pivotal biomarker in SP, demonstrating significant downregulation across healthy controls, SP patients without CD, and SP patients with CD. This expression gradient not only discriminated SP patients from healthy individuals but also stratified CD risk within the SP cohort, positioning miR-143-5p as a promising biomarker for CD risk assessment. The diagnostic value of miR-143-5p was further substantiated by its robust inverse correlations with systemic inflammation markers (WBC, CRP, PCT), cardiac injury indices (LVEF, cTnI), and validated severity scores (APACHE II, SOFA). Elevated WBC, CRP, and PCT levels reflected the acute-phase responses and innate immune activation during SP progression [28, 29]. The observed association between miR-143-5p expression and inflammatory markers suggested its potential involvement in regulating the inflammatory processes of SP. Notably, this correlation was found specifically at the 24-hour post-admission time point, highlighting a critical direction for future research investigating the dynamic changes in miR-143-5p expression patterns across different phases of SP progression. Understanding these temporal dynamics could provide valuable insights into the potential role of miR-143-5p as a time-dependent regulatory molecule in SP pathophysiology. Reduced LVEF (denoting myocardial contractility impairment) and elevated cTnI (indicative of cardiomyocyte injury) delineated distinct interconnected pathways of SP-induced cardiac damage, underscoring the multifactorial pathophysiology of CD [30, 31]. In comparison with cTnI and LVEF, miR-143-5p showed significant advantages in CD. First, the stability in peripheral biofluids ensures consistent miR-143-5p detection [32], unlike fluctuating cTnI (elevated in non-sepsis cardiac stress or reduced by delayed processing) [33]. Our findings indicated that miR-143-5p was upregulated in SP (vs. healthy) and further elevated in CD (vs. SP without CD), which leverages this for reliable diagnosis. Second, cell/disease specificity boosted the accuracy of miR-143-5p for CD diagnosis, unlike nonspecific LVEF (normal in early CD, reduced in other cardiac disorders) [34]. These traits make miR-143-5p a more useful biomarker, filling a sepsis management gap. The integration of APACHE II and SOFA scoring systems provided a robust framework for risk stratification in SP, with APACHE II quantifying acute physiological derangements and SOFA dynamically monitoring SP-induced organ failure, particularly cardiovascular compromise [35]. Notably, SP patients with CD exhibited significantly elevated APACHE II and SOFA scores, reflecting aggravated systemic derangement and organ dysfunction‌, which was consistent with Meng’s study [36]. Furthermore, combined ROC analyses demonstrated that the integration of miR-143-5p with either APACHE II or SOFA scores yielded superior diagnostic performance for CD in SP patients. The strong inverse correlation between miR-143-5p levels and these severity indexes further underscored its association with SP progression‌. Multivariate analysis identified miR-143-5p downregulation as a risk factor for CD in SP, alongside established predictors including reduced LVEF, increased cTnI, and elevated APACHE II/SOFA scores [37–39]. Compared to other miRNAs, including miR-155 (8), miR-21(9), and miR-208a-5p (10), this integrated clinical trials and in vitro cell experiments had verified the consistent expression pattern of miR-143-5p in both human samples and cell models, thereby enhancing our understanding of its potential clinical applications, which might represent a distinct advantage of this work. These findings suggested the potential of miR-143-5p as both a biomarker and therapeutic target in SP-associated myocardial injury.

To further explore the effect of miR-143-5p in the pathological process of SP and SP-induced CD, the macrophage and myocardial cell injury models were conducted by LPS. THP-1 is a well-characterized human monocytic cell line that acquires macrophage-like properties, including the ability to mount robust inflammatory responses to bacterial LPS [40]. Since LPS is a major component of the outer membrane of Gram-negative bacteria and a potent inducer of the systemic inflammatory response syndrome (SIRS) that underlies the pathophysiology of SP [41], LPS-stimulated THP-1 macrophages serve as a widely accepted, relevant, and reproducible in vitro model to mimic the early inflammatory events triggered by bacterial infection in SP. Macrophage polarization critically modulates SP pathophysiology through phenotype switching and inflammatory response regulation [42]‌. Early M1 polarization dominance amplifies pro-inflammatory cytokine storms (e.g., TNF-α, IL-6), exacerbating inflammatory response and organ injury‌, while delayed M2 polarization skewing contributes to immunosuppression and secondary infections [43]. Targeting this dichotomous polarization may rebalance immune homeostasis, attenuating both hyperinflammation and immunosuppressive phases, thus highlighting macrophage plasticity as a therapeutic node to improve SP outcomes by precision immunomodulation. MiR-143-5p has been reported to have a suppressive effect on macrophage M1 polarization and inflammation in abdominal aortic aneurysm, as miR-143-5p overexpression could suppress the expression of CCL20 and then promote the M2 polarization and inhibit the inflammation of macrophage [44]. Our study also confirmed that overexpressed miR-143-5p expression showed a promotive effect on the M2 polarization and anti-inflammatory effect of macrophage, as upregulation of miR-143-5p could suppress the M1 polarization by decreasing M1 polarization marker (iNOS and CD86), elevating M2 polarization marker (Arg-1 and CD206), and suppressing the inflammatory cytokines of TNF-α and IL-6.

SP-associated CD, driven by systemic inflammation and immune dysregulation, is closely linked to cardiomyocyte injury [45, 46]. In LPS-stimulated cardiomyocytes, the observed reduction in cell viability, increased apoptosis, and elevated inflammatory cytokine levels underscored the critical role of myocardial cell damage in SP-related cardiac pathology [47, 48]. Notably, the upregulation of miR-143-5p in this model suggested its potential involvement in cardiomyocyte injury. Functional experiments demonstrated that miR-143-5p overexpression enhanced cardiomyocyte survival, suppressed apoptosis, and attenuated inflammatory responses, highlighting its protective role against LPS-induced cardiomyocyte injury. These findings implied that miR-143-5p may act as a regulatory node, counteracting SP-associated myocardial damage by modulating cellular viability, death signaling, and inflammatory cascades. Targeting miR-143-5p could represent a therapeutic strategy to mitigate SP-associated CD.

Bioinformatic analysis identified a total of 432 overlapping target genes of miR-143-5p, and KEGG pathway analysis revealed their involvement in regulating the mRNA surveillance pathway, p53 signaling pathway, and MAPK signaling pathway. Notably, the relevance of the p53 signaling pathway to SP has been previously validated, as activation of the SIRT1/p53/SLC7A11 signaling pathway was shown to ameliorate ferroptosis in rat cardiomyocytes during sepsis-induced cardiomyopathy [49]. Similarly, the MAPK signaling pathway has been reported to participate in the pathomechanisms of both SP and CD [50–52]. Furthermore, the association between miR-143-5p and MAPK has been experimentally validated in odontoblast differentiation [49, 50], which further supported our prediction of an interaction between miR-143-5p and the MAPK pathway.

It must be admitted that some limitations still existed in this study. First, the relatively small sample size of SP patients may restrict the generalizability of the clinical relevance of miR-143-5p in SP-induced CD, necessitating validation in larger, multicenter cohorts. Second, given the biphasic immune response in sepsis (initial hyperinflammation followed by hypo-inflammation), drawing conclusions based solely on miR-143-5p levels measured 24 h post-admission might limit its clinical interpretability. Nevertheless, our observation that downregulated miR-143-5p expression in SP correlated closely with inflammatory markers (WBC and CRP) suggested miR-143-5p levels may fluctuate with the body’s inflammatory status, a possibility warranting further investigation. Third, while miR-143-5p overexpression demonstrated protective effects in LPS-injured cardiomyocytes, its precise downstream targets and associated signaling pathways in SP remain uncharacterized. Based on our initial exploratory studies, SNX3 was identified by the Target Scan Human as a potential downstream target of miR-143-5p. Previous studies also confirmed that SNX3 is significantly upregulated in SP and patients with cardiac failure [53, 54], reinforcing the role of SNX3 as a promising downstream target of miR-143-5p but also highlighting the necessity of further investigations to validate their regulatory relationship and functional relevance in pathological processes. Potential candidates of miR-143-5p, such as MAPK signaling pathways, also warrant further in vivo or in vitro exploration in SP and SP-associated CD. The lack of mechanistic data linking miR-143-5p to these pathways limited our understanding of its regulatory role. Future studies should conduct bioinformatic predictions and functional validation to delineate the molecular network and the therapeutic potential of miR-143-5p in SP.

## Conclusion

MiR-143-5p served as a diagnostic biomarker for SP (AUC = 0.897) and SP-associated CD (AUC = 0.812), correlating with disease severity and cardiac impairment‌. Its downregulation independently predicted CD risk (OR = 0.100)‌, demonstrating its clinical diagnostic potential for SP and SP-associated CD. Mechanistically, overexpression of miR-143-5p mitigated macrophage inflammation‌, M1 polarization, and cardiomyocyte injury, highlighting its role as a therapeutic target for SP management.‌ In future studies, we plan to expand our sample size, implement multi-center research protocols, and conduct complementary in vivo experiments to further elucidate the mechanistic role of miR-143-5p in SP and SP-associated CD. These approaches will enhance the generalizability of our findings and provide deeper insights into potential therapeutic targets.

## Supplementary Information

Supplementary Material 1.

Supplementary Material 2.

## Data Availability

The datasets used and/or analyzed during the current study are available from the corresponding author on reasonable request.
