# Supplementary figures and images for: MiR-143-5p serves as a diagnostic biomarker in patients with sepsis and regulates sepsis-induced inflammation and cardiac dysfunction
Source: Hereditas. 2025 Dec 10;163:12. doi: 10.1186/s41065-025-00623-0 (PMC12801460; doi:10.1186/s41065-025-00623-0)

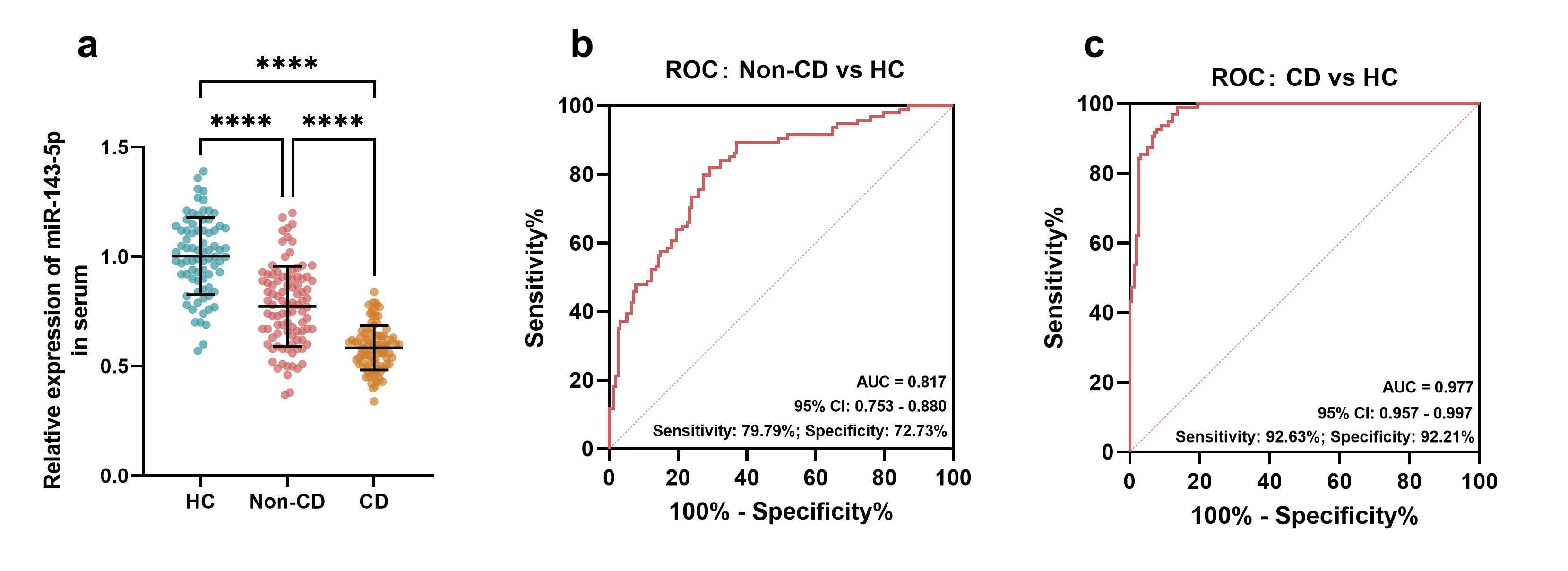

Supplement: Supplementary file 1 — Supplementary Material 1. [file 41065_2025_623_MOESM1_ESM.tif]
