# Supplementary material for: MiR-143-5p serves as a diagnostic biomarker in patients with sepsis and regulates sepsis-induced inflammation and cardiac dysfunction
Source: Hereditas. 2025 Dec 10;163:12. doi: 10.1186/s41065-025-00623-0 (PMC12801460; doi:10.1186/s41065-025-00623-0)
Supplement: Supplementary file 2 — Supplementary Material 2. [file 41065_2025_623_MOESM2_ESM.docx]

Supplementary information 1. The RNA exaction and cDNA synthesis steps.

Total RNA exaction:

Total RNA from serum and cell samples was extracted using Trizol reagent (Sigma-Aldrich, USA) following a standardized protocol optimized for each sample type. For serum samples, 200 μL of serum was mixed with 1 mL of Trizol reagent and vortexed vigorously for 30 seconds, followed by incubation at room temperature for 5 minutes to allow complete dissociation of nucleoprotein complexes. Chloroform (200 μL) was then added, and the mixture was shaken vigorously for 15 seconds and incubated at room temperature for 3 minutes before centrifugation at 12,000 × g for 15 minutes at 4°C. This step separated the solution into three phases: the lower organic phase, an interphase, and the upper aqueous phase containing RNA. The aqueous phase was carefully transferred to a new RNase-free tube, mixed with an equal volume of isopropanol, and incubated at 4°C for 10 minutes to precipitate RNA. After centrifugation at 12,000 × g for 10 minutes at 4°C, the RNA pellet was washed twice with 75% ethanol (prepared with RNase-free water), air-dried for 5-10 minutes, and resuspended in 20 μL of RNase-free water. For cell samples, approximately 1×10⁶ cells were harvested, washed twice with cold PBS, and lysed directly in 1 mL of Trizol reagent by pipetting up and down repeatedly. The subsequent steps (chloroform addition, centrifugation, RNA precipitation, washing, and resuspension) were performed identically to those for serum samples. RNA concentration and purity were assessed using a NanoDrop spectrophotometer, with only samples showing an A260/A280 ratio > 1.8 being used for subsequent analyses.

cDNA synthesis

For cDNA synthesis, 1 μg of total RNA from each sample was reverse-transcribed using the PrimeScript RT Kit (TaKaRa, Japan) in a 20 μL reaction volume. The reaction mixture contained 4 μL of 5× PrimeScript Buffer, 1 μL of PrimeScript RT Enzyme Mix I, 1 μL of RT Primer Mix including Oligo dT Primer and Random 6-mers, 1 μg of RNA template, and RNase-free water to reach the final volume. The reverse transcription protocol consisted of incubation at 37°C for 15 minutes (for cDNA synthesis) followed by 85°C for 5 seconds (to inactivate the reverse transcriptase). The synthesized cDNA was immediately stored at -20°C until use in quantitative real-time PCR analyses.
